# Seroprevalence of SARS-CoV-2 Antibodies in HIV-Infected Patients in Rome, Italy during the COVID-19 Outbreak

**DOI:** 10.3390/diagnostics11071154

**Published:** 2021-06-24

**Authors:** Francesca Lombardi, Rosalba Ricci, Simone Belmonti, Massimiliano Fabbiani, Alberto Borghetti, Gianmaria Baldin, Arturo Ciccullo, Enrica Tamburrini, Elena Visconti, Maurizio Sanguinetti, Simona Di Giambenedetto

**Affiliations:** 1 UOC Malattie Infettive, Fondazione Policlinico Universitario A. Gemelli IRCCS, 00168 Rome, Italy; al.bor86@gmail.com (A.B.); enrica.tamburrini@policlinicogemelli.it (E.T.); elena.visconti@policlinicogemelli.it (E.V.); simona.digiambenedetto@policlinicogemelli.it (S.D.G.); 2 Institute of Microbiology and Virology, Fondazione Policlinico Universitario A. Gemelli IRCCS, 00168 Rome, Italy; rosalba.ricci@policlinicogemelli.it (R.R.); maurizio.sanguinetti@policlinicogemelli.it (M.S.); 3 Dipartimento di Sicurezza e Bioetica Sezione Malattie Infettive, Università Cattolica del Sacro Cuore, 00168 Rome, Italy; simone.belmonti@policlinicogemelli.it; 4 Infectious and Tropical Diseases Unit, Azienda Ospedaliero-Universitaria Senese, 53100 Siena, Italy; massimiliano.fabbiani@gmail.com; 5 Mater Olbia Hospital, 07026 OIbia, Italy; gian.baldin@gmail.com; 6 Gemelli, Molise Hospital, 86100 Campobasso, Italy; arturo.ciccullo@gmail.com

**Keywords:** HIV, SARS-CoV-2, COVID-19, seroprevalence, antiretroviral therapy

## Abstract

Background: this study aimed to determine the proportion of people living with HIV (PLWH) with anti-SARS-CoV-2 IgG antibodies in a large sample from a single HIV referral center in Rome, Italy; the time-frame included both the first and the second wave of the Italian COVID-19 pandemic; Methods: we conducted a cross-sectional study on stored cryopreserved samples from 1 March 2020 to 30 November 2020. Total antibodies against SARS-CoV-2 were preliminarily tested using a chemiluminescent immunoassay. Positive results were re-tested with an ELISA assay as an IgG confirmatory test; Results: overall, 1389 samples were analyzed from 1106 PLWH: 69% males, median age 53 years, 94% on antiretroviral treatment, 93% with HIV-RNA < 50 copies/mL, median CD4 cell count 610 cell/µL. Our analysis revealed a total of n = 8 patients who tested IgG positive during the study period. Seroprevalence was equal to 0% in the first months (March–June); this started to increase in July and reached a maximum rate of 1.59% in October 2020. The overall seroprevalence was 0.72% (8/1106, 95% CI 0.37–1.42). Conclusion: our findings from this setting show a low IgG SARS-CoV-2 prevalence among PLWH as compared to data available from the general population.

## 1. Introduction

Despite the ongoing pandemic spread of severe acute respiratory syndrome coronavirus 2 (SARS-CoV-2), which causes Coronavirus Disease 2019 (COVID-19), the impact of this new coronavirus on people living with HIV (PLWH) is still unclear and data are fragmentary and, at times, controversial [1,2]. In particular a comprehensive understanding of the susceptibility to SARS-CoV-2 infection in PLWH must still be determined; more data are needed to clarify viral transmissibility and to strengthen pandemic prevention and preparedness efforts in this population. Several reports suggested that PLWH, such as immunocompromised subjects, should be considered a vulnerable group because they could have a higher risk of getting SARS-CoV-2, as compared to the general population [3]. Nevertheless, previous large population-based studies found similar [4,5] or lower [6,7] SARS-CoV-2 incidence among PLWH compared with the general population. However, given the high proportion of asymptomatic infections with SARS-CoV-2, incidence estimates from these studies could be biased by differential testing rates among populations [8]. Furthermore, this available data on the SARS-CoV-2 incidence in PLWH derives from RT-PCR positive testing, which detects active infections. On the other hand, serology testing for SARS-CoV-2 antibodies has been recognized as a useful tool for diagnosing both previous and active infection in both symptomatic and asymptomatic individuals. Thus, seroprevalence studies could be used to better estimate the number of individuals who have been infected [9]. Here we present the results of a seroprevalence study measuring IgG antibodies against SARS-CoV-2 in a large sample from a single HIV referral center in Rome, after the beginning of the Italian outbreak (i.e., 21st February 2020), and up until the end of November 2020, which was an extended time-frame including the three phases of the infection in our country.

## 2. Materials and Methods

### 2.1. Study Cohort

This was a monocentric cross-sectional study based on a cohort of PLWH who frequent the healthcare facilities at the Department of Infectious Diseases of the University Hospital ‘Fondazione Policlinico Universitario A. Gemelli IRCCS’—Università Cattolica del Sacro Cuore in Rome, Italy. For research purposes, we have an ongoing observational cohort of HIV positive patients in our center, who routinely attend our hospital outpatient service. All clinical information is prospectively recorded, and the laboratory data are constantly updated in an electronic database. We also systematically collect, and store residual plasma samples obtained from routine viral load measurements (plasma HIV-RNA) from the same outpatients. Thus, for this seroprevalence exploration, we were able to select cryopreserved plasma samples from this pool starting 1 March 2020, and ending 30 November 2020, to assess SARS-CoV-2 IgG seroprevalence. Specifically, we decided to assay all samples available from March to April because only a small number of samples were available due to the reduced flow of patients during the lockdown period. For the remaining periods, we randomly selected a convenient sample size, i.e., 50% of the residual samples per month. We did not establish specific inclusion/exclusion criteria, and we excluded samples only when associated clinical/laboratory data were missing. Thus, for all selected samples we had all appropriate demographical, clinical, laboratory and therapeutic data. The few data that were missing concerned only a small percentage of patients with an unknown risk factor for HIV transmission. 

### 2.2. Evaluation of SARS-CoV-2 Seroprevalence

To assess IgGs against SARS-CoV-2, we first qualitatively screened total antibodies with a chemiluminescent immunoassay, the Atellica^®^ IM SARS-CoV-2 Total assay, using an Atellica^®^ IM Analyzer (Siemens Healthcare, Erlangen, Germany). This assay is directed toward the receptor-binding domain (RBD) in the S1 subunit of the SARS-CoV spike (S) protein. All positive samples were re-tested with a confirmatory ELISA assay, SARS-CoV-2 (Euroimmun, Lübeck, Germany) already in use at our center for diagnostic routines. This assay provides semiquantitative in vitro determination of immunoglobulins classes IgG, using the S1 domain of the Spike protein, including RBD. Sensitivity and specificity for Atellica^®^ IM SARS-CoV-2 Total assays were both 95% and for SARS-CoV-2 Euroimmun ELISA they were 90.3% and 98.5%, respectively. All plasma samples that were confirmed positive for SARS-CoV-2 IgG using the Euroimmunn ELISA test were considered ‘truly’ positive. Based on the random selection design, testing samples from the same subject at different times (months) were considered in the study. 

We estimated an overall seroprevalence and an additional month-by-month seroprevalence as the number of ‘truly’ positive tests/total patients. Seroprevalence was reported as rate and 95% confidence intervals (CI).

### 2.3. Statistical Analysis

For descriptive statistics, continuous variables were summarized as median and interquartile ranges (IQR), and categorical variables were expressed as percentages. We used logistic regression models to explore factors associated with positive SARS-CoV-2 serology in our cohort. The variables analyzed were selected because they were considered factors that are likely to impact susceptibility to SARS-CoV-2 infection in vulnerable patients, such as PLWH. Variables such as age, time on ART, CD4 cell counts, CD4/CD8 ratio, CD4 nadir, and pre-ART HIV-RNA log copies/mL were considered as continuous variables. Variable such as sex, nationality (being Italian), receiving ART, type of ART (triple vs. dual regimen), having HIV-RNA < 50 copies/mL, past AIDS events, and having any comorbidities were binary-coded, whereas risk factor (Homo/bi-sexual, Heterosexual and IVDU), and anchor drug-based regimen (INSTI-based, NNRTI-based and PI-based) were considered as three-level explanatory variables. Statistical significance was defined as a two-sided *p* value of less than 0.05. Data analysis and management were performed using SPSS software version 22.0 (IBM, SPSS, Chicago, IL, USA).

## 3. Results

A total of 1389 samples from 1106 PLWH were analyzed: 69% were male, 83% were Italian, the median age was 53 years (IQR 45–60), the median time from HIV infection diagnosis was 16 years (IQR 8–24); 94% (n = 1038/1106) of patients were on ART (median time 13 years, IQR 6–21) of whom 72% (n = 750/1038) were on a triple regimen with nucleoside reverse transcriptase inhibitors backbone plus an anchor drug (59.5% integrase inhibitor, 31.5% non-nucleoside reverse transcriptase inhibitor, 9% protease inhibitor), 27% (n = 279/1038) were on a dual therapy (mainly 67% integrase inhibitor-based and 16% protease inhibitor-based), and the remaining 0.9% (n = 9/1038) were on different combination regimens. The median CD4 cell count was 610 cell/µL (IQR 436–822), and 93% of patients were virologically suppressed (with HIV-RNA < 50 copies/mL). Table 1 summarizes the main characteristics of the patients according to the date (month) of the plasma samples. Any repeated sample from the same subject occurred in the same month during the study period.

Overall, 13 tests resulted positive for anti-SARS-CoV-2 IgG antibodies using the chemiluminescent immunoassay; of these, eight were confirmed to be IgG positive using the ELISA test. Table 2 reports demographic, clinic and therapeutic characteristics of the eight patients who resulted positive. The probability of seropositivity was the same between men and women (both 50%), median age was 53.4 years, median CD4 cell count 831 cell/μL, and median CD4/CD8 ratio 0.86. Analysis of the clinical records showed that seven patients had a control visit at the same time their blood sample was taken, except for patient 4 who had last visited in November 2019. Only one patient (patient 7) reported recent infection with SARS-CoV-2, which manifested as a mild disease without the need for hospitalization and without any complications. No other patients reported symptoms related to COVID-19 in recent months. Five patients declared that their health was good, and patient 6 reported non-COVID-19 related health issues.

**Figure 1:**
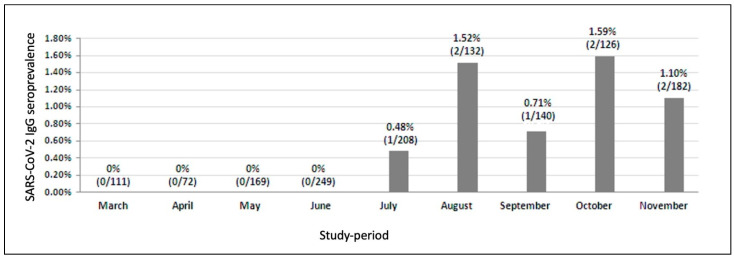
Month by month anti SARS-CoV-2 IgG seroprevalence. Seroprevalence was calculated as the number of ‘truly’ positive tests/total patients × 100.

Overall, our analysis revealed a seroprevalence of 0.72% (n = 8/1106; 95% CI 0.37–1.42). Considering plasma samples month by month, the SARS-Cov-2 IgG/seroprevalence ranged from 0% to 1.59%. Specifically, we did not find any case during March (n = 0/111, 0%; 95% CI 0.00–0.033) April (n = 0/72, 0%; 95% CI 0.00–0.051), May (n = 0/169, 0%; 95% CI 0.00–0.022) and June (n = 0/249, 0%; 95% CI 0.0–0.015); in July and September we found one case respectively [0.48% (n = 1/208), 95% CI 0.085–2.267 and 0.71% (n = 1/140), 95% CI 0.13–3.93]. In August, October and November we found two cases respectively [1.52% (n = 2/132), 95% CI 0.42–5.36; 1.59% (n = 2/126), 95% CI 0.44–5.60 and 1.10% (n = 2/182), 95% CI 0.30–3.92] (Figure 1). In a logistic regression analysis, no demographic or clinical variable was related to a higher risk of infection with SARS-CoV-2; moreover, no relationship with the type of ART received was observed.

## 4. Discussion

The present study was conducted during a period which included the different phases that have characterized the pandemic in Italy. Here, the diffusion of the COVID-19 epidemic can be broken down into three phases. The first one, i.e., the first wave, occurred after the outbreak (i.e., from February to late May), and was characterized by a very rapid spread of the cases and deaths with high territorial concentration in northern Italy [10,11]. During the inter-wave period, which occurred in the summer (i.e., from June to mid-September), the diffusion was initially very contained. However, in late September a growing number of hotspots were identified across the country. In the second wave, from late September, the cases increased exponentially all over the country, and in late November a drop in the incidence of infection was observed [11]. As expected, in our study the month by month SARS-CoV-2 seroprevalence showed that the positivity rate of serological tests increased over time, especially during the second wave, in line with an increase of immunization of the Italian population.

In light of limitations in population-based data, systematic seroprevalence studies are needed to accurately determine attack rates. However, data on SARS-CoV-2 seroprevalence in PLWH are presently very scarce. In a study of 500 patients from a single HIV-center in Munich, Germany, between May and July 2020, the authors stated that SARS-CoV-2 IgG seroprevalence, which was 1.5%, did not significantly differ from that reported in the general population in similar “hot-spot” areas [12].

Another analysis conducted by Papalini et al. in May 2020 on 270 HIV-positive patients at the Diseases Clinic of Perugia Teaching Hospital in the Umbrian area of Central Italy showed no serologically confirmed infection with SARS-CoV-2 versus a 4% IgG seroprevalence, which was found in a parallel HIV-negative control group [13].

Furthermore, a recent systematic seroprevalence study from Spinelli et al. reported that PLWH had approximately two times lower IgG seroprevalence of SARS-CoV-2 than people without HIV in an urban health-care system located in San Francisco, US, over a 3-months period [14].

We can observe that the IgG seroprevalence of SARS-CoV-2 infection found in the PLWH in our study, relatively to the period after the first pandemic wave in early 2020, appears to be lower, even if not dramatically, as compared to the results of a study on SARS-CoV-2 IgG seroprevalence carried out by the Italian Institute of Statistics (ISTAT) on the general population between 25th May and 15th July 2020 [15]. In fact, in this survey the percentage in the Lazio region of Italy, which includes the Rome area, was estimated to be as high as 1%. We found a seroprevalence of 0% in PLWH in the same period. Overall, in our study, the positivity rate of PLWH was markedly lower when compared with the SARS-CoV-2 IgG seroprevalence data obtained in the Vatican City (Rome) general population in a different study [16], which considered the period June 2020-November 2020, and that was also analyzed in our study. Indeed, the percentages of positive cases among individuals from the Vatican City enclave during the inter-wave period varied over time, i.e., it was 5.97% in June, 1% in July and 3.81% in August (vs. 0%, 0.48% and 1.52%, respectively in our PLWH cohort) and during the second wave it was 3.11% in September, 2.06% in October and 7.66% in November (vs. 0.71%, 1.59% and 1.10%, respectively). Thus, the overall infection rates in PLWH in our study appear to be lower than comparative data from the general population from the same Rome area in the corresponding period. Moreover, other available seroprevalence data on the Italian general population from regions with a similar SARS-CoV-2 endemicity during the first pandemic phase showed a higher positivity rate compared to that of the PLWH in our study [17].

We can conclude that our findings from this setting appear to be consistent with the findings from recent IgG seroprevalence studies [13,14], and they support previous population-based analysis [6,7]. Spinelli et al. speculated that PLWHs could have been vigilant because they perceived themselves to be at higher risk, or additional services available to PLWH through local Care programs might have facilitated the ability of PLWH to shelter in place [14]. On the other hand, it was previously postulated that, in PLWH, as in the general population, other non-HIV-related variables, such as work activities or adherence to social-distancing procedures, might have been prominent in determining the risk of infection. [18,19]. In light of our results and the increasing evidence from different contexts, we also think that a systematic analysis of the potential determinants of a low susceptibility to SARS-CoV-2 coinfection among PLWH, including a potential protective role of ART, is warranted, and it should be a focus of future investigations. Since anti-HIV drugs have been proposed as possibly being effective against SARS-CoV-2, some authors have hypothesized that ART, and in particular protease inhibitors, could have a protective effect on SARS-CoV-2 infection [20]. Furthermore, several reports have indicated the use of specific antiretrovirals as potentially protective against the risk of infection with SARS-CoV-2, given its reported effect against SARS-CoV-2 polymerase [21,22,23]. However, in our study we failed to identify an association between the type of ART-regimen or other risk factors and evidence of infection with SARS-CoV-2 in PLWH, probably due to the overall low number of events. All patients who resulted IgG positive were on effective ART, with virological suppression, a high median CD4 count and with a median CD4/CD8 ratio that could be superimposed on the remaining HIV+/SARS-CoV-2 negative subjects. Nevertheless, it is interesting to note that in our study the median age of PLWH who resulted SARS-CoV-2 IgG positive was 51.4 years (IQR 39.5–60.0), which is about a decade lower than the median age observed in the general population with COVID-19 in Italy during the outbreak [24]. Many studies from different contexts have also reported the same phenomenon, which has been associated with a possible accelerated and/or accentuated aging process experienced by PLWH [2].

One strength of this study is that we estimated the seroprevalence rate by considering as ‘truly’ positive only samples which tested positive on both assays employed. Indeed, this double approach substantially increases the positive predictive value of the laboratory result, which is lower when using only one assay in a context where the prevalence in a population is low and it decreases the number of false positive results [25]. On the other hand, a negative test result does not rule out current or previous COVID-19 infection, because it is known that it takes at least 7–14 days to produce a measurable antibody response, and some individuals could not produce a sufficient antibody response at all [26]. A limitation of our study is that we could not compare our population with a sample of sex/age matched HIV-uninfected patients. Unfortunately, prevalence data on the general population are scarce and do not seem to show differences in seroprevalence according to gender. Regarding age, the general Italian data show a lower prevalence of SARS-CoV-2 infection in those aged 0–5 years or >85 years, two age strata that were not included in our sample of HIV-infected patients. Indeed, no differences in prevalence were observed in the other age strata [15]. Therefore, based on these observations, comparing our data with those of the general population might allow us to draw some conclusions, although there is no certainty that the two populations are sex/age matched. The limits of our analysis include the fact that it was focused on the cumulative seroprevalence of anti-SARS-CoV-2 antibodies without differentiating between IgM and IgG. Moreover, we did not confirm IgG positivity against SARS-CoV-2 with the result of a nasopharyngeal RT-PCR test and thus we were unable to differentiate between active and past infections; however, at the time of sampling no patient reported symptoms related to COVID-19. Furthermore, our results can be considered as representative of the Italian HIV-infected population engaged in outpatient care facilities in central Italy; most of these are currently on ART and virally suppressed, as stated in the latest report [27]. Thus, further evaluations are certainly needed in the heterogeneous community of PLWH to analyze cohorts with a different viro-immunological profile. Despite these limitations, to the best of our knowledge, this is the largest amount of data that has been presented on the seroprevalence of SARS-CoV-2 infection in PLWH. Although more comparative data are required, our findings from this setting show a low IgG SARS-CoV-2 prevalence among PLWH as compared to the data available from the general population. Our results represent a novel important contribution regarding the debated potential contribution of HIV and/or ART to the risk of SARSCoV-2 co-infection in PLWH.

## Figures and Tables

**Table 1:** Characteristics of the entire study samples (n = 1389) from HIV-infected patients (n = 1106) according to the month.

**(A). Demographic Characteristics**										
	**March 2021 n = 111**	**April 2021 n = 72**	**May 2021n = 169**	**June 2021 n = 249**	**July 2021 n = 208**	**August 2021 n = 132**	**September 2021 n = 140**	**October 2021 n = 126**	**November 2021 n = 182**	**PLWH Population n = 1106**
Gender, n (%)										
Male	74 (66.7)	51 (70.8)	114 (67.5)	169 (67.9)	135 (64.9)	85 (64.4)	102 (72.9)	91 (72.2)	131 (72.0)	760 (68.7)
Age, years, (IQR)	54.1 (45.2–59.3)	48.5 (36.6–58.9)	51.9 (44.7–60.1)	53.3 (46.4–58.9)	55.4 (43.8–60.4)	52.2 (42.1–56.4)	53.2 (43.4–59.7)	54.0 (44.6–63.6)	53.1 (46.1–59.4)	52.9 (44.8–59.3)
Italian, n (%)	88 (79.3)	59 (81.9)	139 (82.2)	198 (79.5)	165 (79.3)	110 (83.3)	111 (79.3)	108 (85.7)	159 (87.4)	912 (82.5)
Risk factor, n (%)										
Homo/bi-sexual	34 (30.6)	20 (27.8)	60 (35.5)	72 (28.9)	61 (29.3)	40 (30.3)	53 (37.9)	43 (34.1)	70 (38.5)	362 (32.6)
Heterosexual	40 (36.0)	16 (22.2)	61 (36.1)	111 (44.6)	77 (37.0)	61 (46.2)	48 (34.3)	50 (39.7)	71 (39.0)	431(38.5)
IVDU	15 (13.5)	11 (15.3)	21 12.4)	30 (12.0)	31 (14.9)	20 (15.2)	22 (15.7)	9 (7.1)	18 (9.9)	146 (13.2)
Time since HIV diagnosis, years, median (IQR)	15.6 (7.6–23.8)	9.8 (1.18–22.4)	14.7 (6.5–22.4)	16.6 (7.5–23.9)	16.5 (7.4–25.8)	16.2 (8.9–26.1)	16.0 (6.3–24.3)	13.0 (6.5–22.4)	18.7 (11.5–24.3)	15.9 (7.6–23.7)
Pre-ART HIV-RNA, log_10_ copies/mL, median (IQR)	5.0 (4.1–5.4)	4.9 (3.8–5.5)	5.0 (4.2–5.4)	4.9 (4.1–5.4)	4.9 (4.1–5.4)	4.9 (4.4–5.4)	4.9 (4.5–5.4)	4.9 (4.1–5.6)	4.9 (4.2–5.5)	4.9 (4.2–5.4)
CD4 cell count nadir, cells/mm^3^, median (IQR)	205 (67–336)	171 (36–320)	170 (43–327)	173 (50–291)	200 (85–322)	201 (74–285)	125 (41–277)	159 (36–353)	176 (50–299)	190 (56–315)
CD4 count, cells/mm^3^, median (IQR)	577 (477–773)	487 (275–730)	615 (355–786)	559 (379–781)	619 (463–859)	630 (486–865)	587 (436–830)	603 (434–871)	676 (474–844)	610 (436–822)
CD4/CD8 ratio, median (IQR)	0.79 (0.52–1.13)	0.74 (0.39–1.08)	0.82 (0.53–1.25)	0.85 (0.50–1.18)	0.89 (0.61–1.32)	0.88 (0.59–1.24)	0.85 (0.62–1.16)	0.84 (0.55–1.18)	0.90 (0.59–1.27)	0.87 (0.57–1.23)
HIV-RNA <50 copies/mL, n (%)	100 (90.1)	56 (77.8)	154 (91.1)	238 (95.6)	192 (92.3)	121 (91.7)	126 (90.0)	114 (90.5)	173 (95.1)	1029 (93.0)
Past Aids-defining events (CDC stage C), n (%)	32 (28.8)	18 (25)	50 (29.6)	87 (34.9)	55 (26.4)	35 (26.5)	48 (34.3)	49 (38.9)	58 (31.9)	335 (30.3)
**(B). Clinical Characteristics**										
	**March 2021 n = 111**	**April 2021 n = 72**	**May 2021 n = 169**	**June 2021 n = 249**	**July 2021 n = 208**	**August 2021 n = 132**	**September 2021 n = 140**	**October 2021 n = 126**	**November 2021 n = 182**	**PLWH Population n = 1106**
Patients with at least one comorbidity, n (%)	47 (42.3)	22 (30.6)	77 (45.6)	124 (49.8)	94 (45.2)	51 (38.6)	72 (51.4)	58 (46.0)	90 (49.5)	498 (45.0)
Dyslipidemia, n (%)	4 (3.6)	2 (2.8)	10 (5.9)	13 (5.2)	3 (5.2)	3 (2.3	8 (5.7)	7 (5.6)	6 (3.3)	44 (4.0)
Hypertension, n (%)	11 (9.9)	2 (2.8)	19 (11.2)	30 (12.0)	24 (11.5)	15 (11.4)	12 (8.6)	15 (11.9)	24 (13.2)	118 (10.7)
HCV co-infection, n (%)	23 (20.7)	10 (13.9)	27 (16.0)	39 (15.7)	43 (20.7)	26 (19.7)	33 (23.6)	12 (9.5)	33 (18.1)	193 (17.5)
HBV co-infection, n (%)	2 (1.8)	1 (1.4)	5 (3.0)	9 (3.6)	4 (1.9)	3 (2.3)	6 (4.3)	5 (4.0)	5 (2.7)	30 (2.7)
Diabetes mellitus, n (%)	3 (2.07)	2 (2.8)	4 (2.4)	5 (2.0)	2 (1.0)	1 (0.8)	5 (3.6)	3 (2.4)	3 (1.6)	22 (2.0)
Renal diseases, n (%)	3 (2.7)	1 (1.4)	2 (1.2)	9 (3.6)	7 (3.4)	2 (1.5)	3 (2.1)	5 (4.0)	5 (2.7)	29 (2.6)
Cardiovascular diseases, n (%)	11 (9.9)	8 (11.1)	21 (12.4)	38 (15.3)	27 (13.0)	9 (6.8)	15 (10.7)	17 (13.5)	26 (14.3)	130 (11.8)
History of neoplasms, n (%)	18 (16.2)	6 (8.3)	26 (15.4)	54 (21.7)	30 (14.4)	14 (10.6)	27 (19.3)	22 (17.5)	30 (16.5)	174 (15.7)
**(C). Therapeutic Characteristics**										
	**March 2021 n = 111**	**April 2021 n = 72**	**May 2021 n = 169**	**June 2021 n = 249**	**July 2021 n = 208**	**August 2021 n = 132**	**September 2021 n = 140**	**October 2021 n = 126**	**November 2021 n = 182**	**PLWH Population n = 1106**
Currently on ART, n (%)	97 (87.4)	52 (72.2)	161 (95.3)	237 (95.2)	200 (96.2)	130 (98.5)	135 (96.4)	121 (96.0)	178 (97.8)	1038 (93.9)
Time on ART, years, median (IQR)	12.1 (5.5–20.5)	9.7 (0.68–17.6)	11.2 (5.7–21.0)	14.0 (6.5–22.1)	11.9 (5.3–20.9)	14.5 (7.0–21.6)	12.5 (5.9–22.0)	11.1 (5.6–21.3)	15.8 (9.4–22.2)	12.7 (6.4–21.2)
Type of ARTs, n (% = n/currently on ART)										
Triple regimen	71 (73.2)	45 (86.5)	113 (70.2)	176 (74.3)	155 (77.5)	94 (72.3)	96 (71.1)	82 (67.8)	116 (65.2)	750 (67.8)
Dual regimen	25 (25.8)	7 (13.5)	48 (29.8)	58 (24.5)	41 (20.5)	36 (27.7)	39 (28.9)	38 (31.4)	60 (33.7)	279 (25.2)
Other combinations	1 (1.0)	0 (0.0)	0 (0.0)	3 (1.3)	4 (2.0)	0 (0.0)	0 (0.0)	1 (0.8)	2 (1.1)	9 (0.80)
Triple regimen based, n (%)										
INSTI-based	47 (66.2)	30 (66.7)	67 (59.3)	92 (52.3)	100 (64.5)	53 (56.4)	67 (69.8)	57 (69.5)	74 (63.8)	446 (59.5)
PI-based	6 (8.5)	4 (8.9)	12 (10.6)	18 (10.2)	11 (7.1)	8 (8.5)	11 (11.5)	7 (8.5)	8 (6.9)	68 (9.1)
NNRTI-based	18 (25.4)	11 (24.4)	34 (30.1)	66 (37.5)	44 (28.4)	33 (35.1)	18 (18.8)	18 (22.0)	34 (29.3)	236 (31.5)
Dual regimen based, n (%)										
INSTI-based	17 (68.0)	6 (85.7)	34 (70.8)	40 (69.0)	29 (70.7)	19 (52.8)	23 (59.0)	27 (71.1)	43 (71.7)	186 (66.7)
PI-based	2 (8.0)	0 (0.0)	6 (12.5)	9 (15.5)	6 (14.6)	6 (16.7)	6 (15.4)	6 (15.8)	8 (13.3)	44 (15.8)
Other dual	6 (24.0)	1 (14.3)	8 (16.7)	9 (15.5)	6 (14.6)	11 (30.6)	10 (25.6)	5 (13.2)	9 (15.0)	49 (17.6)

Abbreviations: IQR, interquartile range; IVDU, Intravenous drug users; ART, Antiretroviral Therapy; INSTI, Integrase strand transfer inhibitors; PI, Proteinase inhibitor; NNRTI, Non-Nucleoside Reverse Transcriptase Inhibitor; HCV, Hepatitis C virus; HBV, Hepatitis B virus.

**Table 2:** Demographics, clinical and therapeutic characteristics of the eight SARS-Cov-2 IgG seropositive subjects.

Pz	1	2	3	4	5	6	7	8
Month	July	August	August	September	October	October	November	November
Gender	F	F	F	F	M	M	M	M
Age, years	61.5	55.5	39.5	47.9	39.6	66.5	51.4	58.5
Italian	Yes	Yes	Yes	No, Uganda	Yes	Yes	Yes	Yes
Risk factor	Heterosexual	Heterosexual	Heterosexual	Heterosexual	Unknown	Heterosexual	Homo/bi-sexual	Homo/bi-sexual
Time since HIV diagnosis, years	27.1	21.0	24.2	14.0	3.0	22.6	12.7	24.3
Currently on ART	Yes	Yes	Yes	Yes	Yes	Yes	Yes	Yes
Type of ARTs	Triple	Triple	Triple	Dual	Dual	Dual	Triple	Dual
	INSTI-based	INSTI-based	NN-based	PI-based	INSTI-based	NN/INSTI	NN-based	PI-based
	3TC,ABC,DTG	FTC,TAF,RGV	FTC,TDF,EFV	3TC,ATV,cob	3TC,DTG	RPV,DTG	FTC,TAF,RPV	3TC,DRV,RTV
Time on ART, years	27.1	20.8	23.8	13.9	3.0	12.3	11.8	20.9
Pre-ART HIV-RNA, Log_10_ cps/mL	4.15	5.24	5.70	5.70	4.54	5.20	4.7	5.70
CD4 cell count nadir, cells/mm^3^	206	298	36	119	390	326	258	328
CD4 count, cells/mm^3^	524	1304	1095	783	859	885	423	804
CD8 count, cells/mm^3^	620	1094	459	899	1205	1415	673	555
CD4/CD8 ratio	0.84	1.19	2.39	0.87	0.71	0.63	0.62	1.45
HIV-RNA, copies/mL	0	18	0	0	0	0	0	0
Past Aids-defining events (CDC stage C)	/	/	Yes	Yes	/	/	/	/
Dyslipidemia	/	/	/	/	/	/	/	/
Hypertension	/	/	/	/	/	/	/	Yes
HCV/HBV co-infection	/	/	/	/	/	/	/	/
Diabetes mellitus	/	/	/	/	/	/	/	/
Renal diseases	/	/	/	/	/	/	/	/
Cardiovascular diseases	/	/	/	/	/	Yes	/	/
History of neoplasms	/	/	/	Yes	/	/	/	/
SARS-CoV-2 IgG	1.2	1.5	1	4.5	1.1	3.3	1.8	2.2

Abbreviations: 3TC, lamivudine; ABC, abacavir; DTG, dolutegravir; FTC, emtricitabine; TDF, tenofovir disoproxil fumarate; TAF, tenofovir alafenamide fumarate; EFV, efavirenz; ATV, atazanavir; DRV, darunavir, RPV, rilpivirine; RTV, ritonavir; cob, cobicistat.

## Data Availability

All the data used in this study will be made available upon request.
